# Early Diagnosis of Extrahepatic Biliary Atresia in an Open-Access Medical System

**DOI:** 10.1371/journal.pone.0049643

**Published:** 2012-11-20

**Authors:** Justin Hollon, Matilda Eide, Gregory Gorman

**Affiliations:** 1 Department of Pediatrics, Uniformed Services University of the Health Sciences, Bethesda, Maryland, United States of America; 2 Division of Pediatric Gastroenterology and Nutrition, Johns Hopkins University School of Medicine, Baltimore, Maryland, United States of America; 3 Department of Pediatrics, Walter Reed National Military Medical Center, Bethesda, Maryland, United States of America; California Pacific Medical Center Research Institute, United States of America

## Abstract

**Introduction:**

Biliary atresia (BA) is the most common cause of cholestatic jaundice in infancy. Early diagnosis and surgical management, ideally before 60 days of age, result in improved outcomes. We aimed to determine the age at diagnosis of BA in the Military Health System (MHS) and to compare the age at diagnosis by access to care models. We hypothesized that children with BA receiving primary care in military facilities have an earlier age at diagnosis due to decreased economic and access barriers.

**Methods:**

Data for all Tricare enrollees born in fiscal years 2004–2008 with a diagnosis of BA were extracted from MHS databases. Non-parametric tests, Kaplan-Meier curves and log rank tests compared differences in age at diagnosis by type of primary care facility, gender, prematurity and presence of additional anomalies.

**Results:**

64 subjects were identified within the five year period. Median age at diagnosis was 40 days [range 1–189], with 67% diagnosed by 60 days and 80% by 90 days. 45 (70%) received civilian primary care within the MHS. There was no difference in the median age at diagnosis between subjects in the MHS with civilian primary care vs. military primary care (37 days [1–188] vs. 46 days [1–189]; p = 0.58).

**Conclusion:**

In the MHS, two-thirds of infants with biliary atresia are diagnosed prior to 60 days of life. Gender, prematurity or presence of additional anomalies do not affect the timing of diagnosis. Civilian and military primary care models within the MHS make timely diagnoses of biliary atresia at equivalent rates.

## Introduction

Cholestatic jaundice in the newborn period is a marker of hepatobiliary dysfunction that necessitates a timely work-up and diagnosis. The most common cause of cholestatic jaundice in the first months of life, and the major indication for pediatric liver transplantation, is biliary atresia (BA), a condition characterized by an inflammatory obliteration of the extrahepatic biliary system. The incidence of BA in the US is 1 in 12,000–14,000 live births [1], [2]. Infants may appear healthy with normal birthweight and appropriate weight gain until this sclerosing process eventually leads to signs of progressive disease, such as hepatomegaly, ascites, wasting and coagulopathy. Early diagnosis is essential in order to maximize the survival of the infant’s native liver [3]. Early detection of BA permits surgical repair by the Kasai portoenterostomy, which can restore bile flow, prevent further biliary cirrhosis and worsening liver disease and prevent or at least delay liver transplantation [4]. It has been well established that the earlier the Kasai procedure is performed, the better the outcome, with the goal to perform this surgical procedure before 45 to 60 days of age [5]–[7].

Access to medical care before this 45–60 day mark is critical to making the diagnosis of biliary atresia. However, the relationship between the timing of BA diagnosis and access to medical care has not been studied. This may be particularly important in the U.S. where the routine pediatric well-baby schedule does not include a scheduled visit between 2 and 8 weeks of age. Economics and overall access may play a significant role in influencing whether parents seek care between these standard scheduled visits.

The U.S. Military Health System provides an open access medical system to all of its active duty members and their family members free of charge at its Military Treatment Facilities (MTF). MTFs are typically located on or near a military base and capabilities range from acute care clinics to tertiary care medical centers. Beneficiaries may also opt into several health plans administered by the MHS that allow them to receive care at civilian facilities at a small financial cost, for instance through a managed care plan that requires copayments for each visit. With over 9.3 million beneficiaries in a system that theoretically eliminates economic barriers to access, the MHS has served as the study population in several studies evaluating how ready access to healthcare affects the prevalence, stage at diagnosis and predictive survival of specific diseases [8]–[10]. However, none have looked at diseases affecting infants. In this study, we aim to see if those patients with a diagnosis of biliary atresia who receive care at military treatment facilities have a younger age of diagnosis than those who receive their primary care at civilian facilities.

## Methods

This was a minimal risk retrospective study approved by the Uniformed Services University Institutional Review Board. Data were analyzed anonymously using a pre-existing database of clinical, financial and beneficiary information for MHS operations. Included subjects were identified using the Standard Ambulatory Data Record (SADR), Standard Inpatient Data Record (SIDR) and outpatient and inpatient purchased care claims databases. These databases, collectively called the M2 Database, contain health care data, such as diagnostic coding, for all military beneficiaries worldwide and include data from both military and civilian treatment facilities. Using the International Classification of Diseases, 9th Revision (ICD-9) code for BA (751.61), all subjects born in fiscal years (FY) 2004–2008 who had BA diagnosis prior to 30 May 2009 were included. All military and civilian facility visits for these subjects were extracted from the M2 database from FY 2004 to 30 May 2009. In order to capture only those patients born within the MHS, only those seen within the MHS prior to 21 days of life for any reason were included. To adjust for so-called ‘rule-out coding’, in which patients are coded at an outpatient visit with the possible or suspected diagnosis, rather than the symptom for which further testing or consultation was ordered (e.g. cholestasis), subjects needed at least one inpatient admission or two outpatient visits coded for BA to be considered to carry a diagnosis of BA and remain within the study. This inclusion criteria is identical to that used by the Department of Defense Birth and Infant Health Registry in the 2008 Congenital Malformation Surveillance Report [11], and recognizes that, as a chronic condition, true BA patients continue to necessitate follow-up care and monitoring irrespective of surgical repair. The age at first diagnosis was defined as the earliest occurrence of an inpatient or outpatient encounter coded as BA. Any subjects with an age of first diagnosis greater than 365 days of life were excluded as presumed misdiagnoses or as subjects with missing data.

Subjects’ place of primary care was classified as military or civilian by the type of facility providing the majority of primary care visits prior to BA diagnosis. Subjects were classified as premature if they were diagnosed with ICD-9 codes 765.21–765.28 (<37 weeks of gestation). Additional congenital anomalies were assessed by searching subjects’ records for ICD-9 codes 740.0–759.9. As the ductus arteriosus may physiologically be open at birth and, furthermore, some mild persistent patency may be associated with immaturity at birth, patent ductus arteriosus (ICD-9 747.0) was not included as a congenital anomaly for purposes of comparison. Non-parametric tests, Kaplan-Meier curves and log rank tests compared differences in the ages at diagnosis by type of primary care facility, as well as by gender, gestational age, and presence of additional anomalies. As race data were not available for the majority of subjects it was not included as data for comparison.

Multivariate proportional hazards modeling was performed to determine the effect of confounding variables. Tests of proportional-hazards assumptions were performed using Schoenfeld residuals. Post-hoc power analysis to determine the minimum detectable difference was performed to gauge the chance of a type II error using a power of 0.8. Significance was defined as p-values<0.05. All analyses were conducted with Stata Intercooled 10 (Statacorp, College Station, TX).

## Results

**Figure 1:**
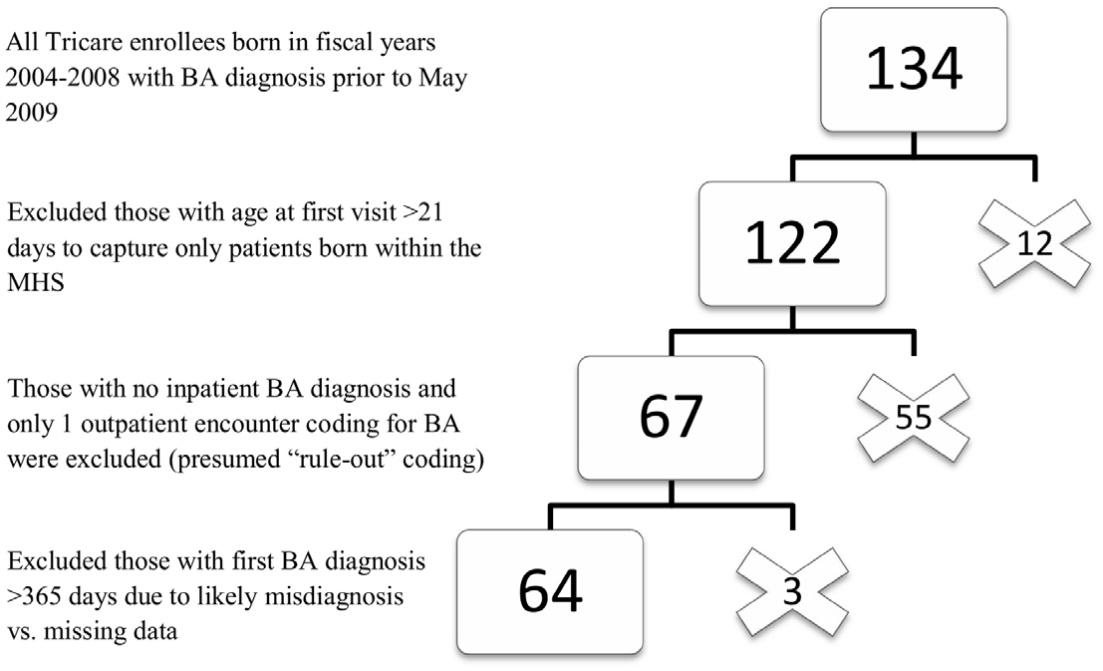
Exclusion algorithm for identification of biliary atresia patients in the MHS. MHS = Military Health System; BA = Biliary Atresia.

134 children with ICD-9 code 751.61 for BA were born in the five-year study period of FY 2004–2008. Of these, 64 subjects met the inclusion criteria. Details of the exclusion process are shown in Figure 1. Table 1 shows the characteristics of included subjects and their distribution by FY. Of the 64 subjects, 17 (27%) were identified as having additional congenital anomalies. Table 2 shows the occurrence of additional anomalies, with the majority (14 of the 17; 82%) having non-PDA congenital heart disease. The most prevalent BA-associated congenital heart defect in this population was a secundum ASD, with 11 of the 14 (79%) having this diagnosis. Just over half (9 of the 17) of these subjects had non-BA gastrointestinal disease, such as small intestinal atresia. One subject had spina bifida and one subject had trisomy 13.

**Table 1:** Demographics and distribution by fiscal year (FY) of all 64 biliary atresia born in the military health system FY 2004–2008.

Variables	n (%)
Civilian primary care	45 (70)
Male	38 (59)
Premature	15 (23)
Additional anomalies	17 (27)
Born in FY:	
2004	21 (33)
2005	13 (20)
2006	15 (23)
2007	8 (13)
2008	7 (11)

**Table 2:** Occurrence of additional congenital anomalies among the 64 biliary atresia patients.

	n (% of total study population)
Additional congenital anomalies	17 (27)
Non-PDA congenital heart disease	14 (22)
Secundum ASD	11 (17)
Tetralogy of Fallot	1 (2)
Coarctation of aorta	1 (2)
Anomalous pulmonary artery	1 (2)
Ebstein’s anomaly	1 (2)
Non-BA Gastrointestinal Disease	9 (14)
Small intestinal atresia	4 (6)
Malrotation/volvulus	3 (5)
Hirschsprung’s	2 (3)
Abdominal wall defect	1 (2)
Pyloric stenosis	1 (2)
Trisomy 13	1(2)
Spina bifida	1(2)

PDA = Patent Ductus Arteriosus; ASD = Atrial Septal Defect; BA = Biliary Atresia.

**Figure 2:**
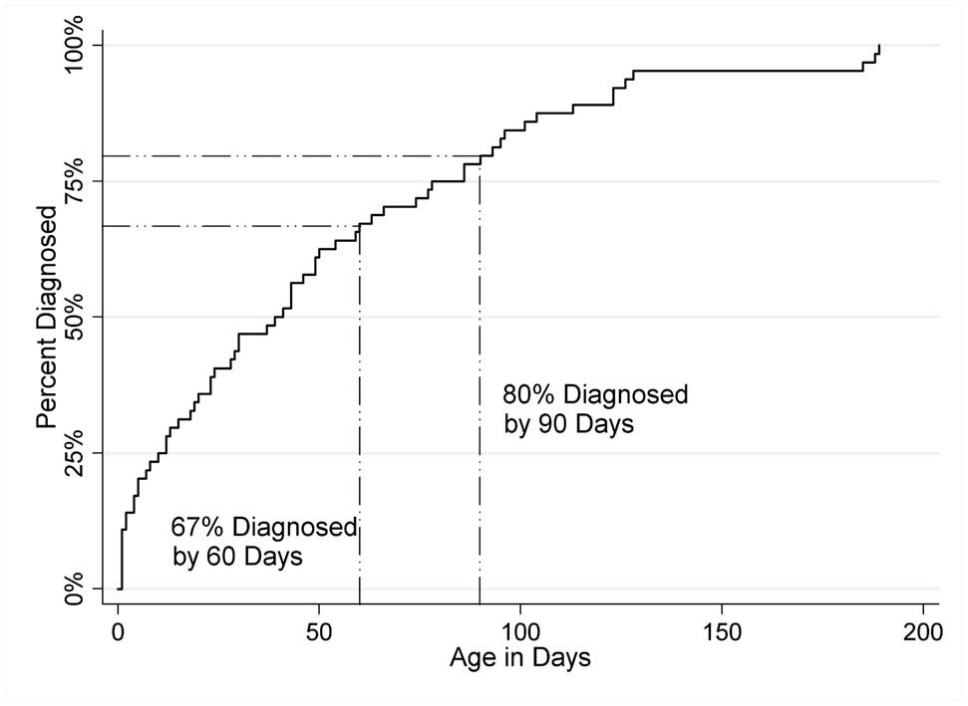
Cumulative prevalence of documented biliary atresia within the Military Health System by age at diagnosis.

The median age of diagnosis of BA in the MHS was 40 days (range of 1–189), with 67% of subjects diagnosed by 60 days of age and 80% diagnosed by 90 days of age (Figure 2). There was no significant difference in median age at diagnosis between those with civilian primary care and those followed at military treatment facilities (37 days [1–188] vs. 46 days [1–189]; p = 0.58). Likewise, there were no differences in median age at diagnosis when examining other comparison groups. The median age at diagnosis for males vs. females was 30 days [1–188] vs. 49 days [1–189]; p = 0.075. Premature infants were diagnosed at a median age of 37 days [1–128] compared to term infants who had a median age of diagnosis of 41 days [1–189]; p = 1. Subjects who had congenital anomalies in addition to BA had a median age of diagnosis of 30 days [1–189] compared to 43 days [1–188] for those with biliary atresia alone (p = 0.57). Median age of diagnosis by FY was 43 [1–188] for 2004, 50 [2–126] for 2005, 23 [1–189] for 2006, 31 [2–128] for 2007 and 29 [1–123] for 2008 (p = 0.67).

Post-hoc power analysis determined that the minimum detectable hazard ratio for the sample was 0.5.

**Figure 3:**
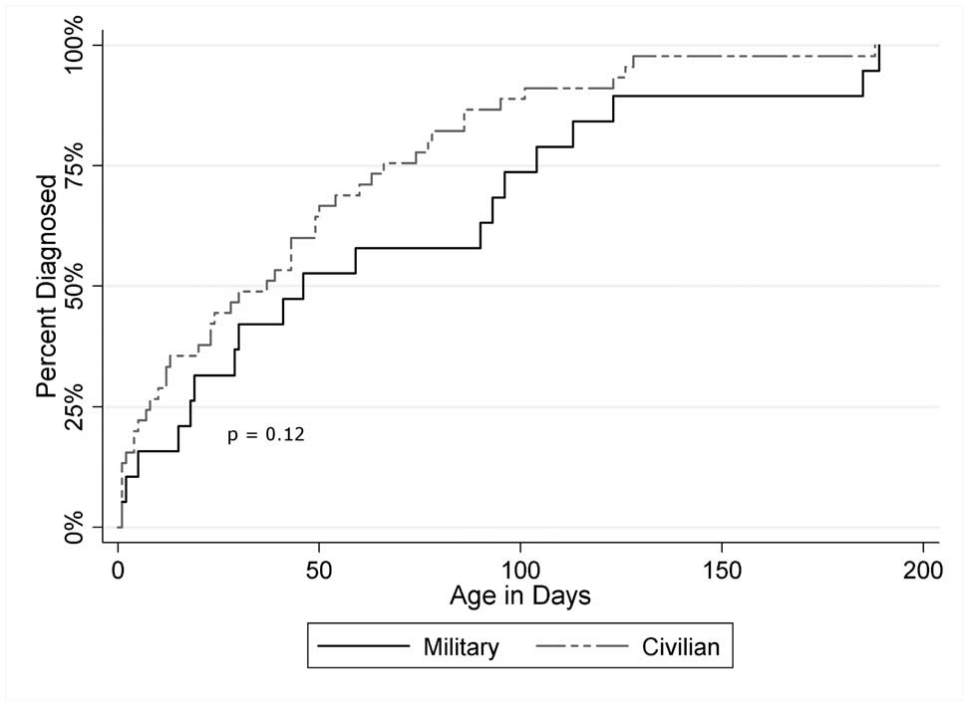
Cumulative prevalence of documented biliary atresia within the Military Health System by primary care model.

Log-rank tests of cumulative time to diagnosis showed no significant difference by type of primary care facility (p = 0.12) (Figure 3), gender (p = 0.19), prematurity (p = 0.95), or presence of additional anomalies (p = 0.86).

In multivariate proportional hazards modeling of the effect of the primary care model adjusted for prematurity, gender, and the presence of additional congenital anomalies, there was no significance in earlier diagnosis in care provided by civilian vs. military providers (HR 1.45 [95%CI 0.81–2.6; p = 0.21]). Likewise, there was no significant difference in time to diagnosis by gender or the presence of anomalies in multivariate models. Hazard ratio estimates of the model are shown in Table 3.

**Table 3:** Hazard ratios of time to diagnosis of biliary atresia by type of primary care model within the Military Health System.

	HR (95% CI)	p-value
Unadjusted Model		
Civilian vs. Military PC	1.48 [0.83–2.63]	0.19
Adjusted Model		
Civilian vs. Military PC	1.46 [0.81–2.57]	0.21
Male vs. Female	1.45 [0.85–2.50]	0.18
Prematurity	1.04 [0.56–1.95]	0.90
Anomalies	0.87 [0.46–1.66]	0.68

Test of proportional hazards assumption p-value = 0.78.

PC = Primary Care.

## Discussion

Early detection of BA permits surgical repair by the Kasai portoenterostomy. In the industrial world, the median age at Kasai operation is between 54 and 69 days of age [12]. Unfortunately, even with a successful Kasai, more than 70% of children eventually develop cirrhosis and require liver transplantation prior to adulthood, but much of this depends on age of surgery [13]. In a retrospective analysis of 349 Canadian patients treated with the Kasai procedure, those who had surgery at<than 30 days of age had a 52% survival of the native liver vs. a 21% survival rate for those who underwent surgery at>90 days of age [13].

For the primary care clinician, improving the age of diagnosis is akin to improving the prognosis of the Kasai. To this aim, parent education on the signs and symptoms of BA, particularly acholic stools, has been a major focus in many countries [14], [15]. In Taiwan, an infant stool color card is sent home with every neonate to educate parents on the importance of acholic stools and increase the rate of early self-referral. A recent study credits this card program for increasing the rate of detection before 60 days of age to 72.5% in 2004 (start of program) and 97.1% in 2005, with subsequent age at Kasai before 60 days of age improving from 23.3% (1976–1989 historical data) to 60% in 2004 and 74.3% in 2005 [14]. What’s not mentioned in this study is Taiwan’s establishment of National Health Insurance (NHI) in 1995, prior to which 41% of the population was uninsured [16]. With a concomitant dramatic increase in childhood immunizations since NHI implementation [17], one must wonder if greater access to care in this population has also strongly contributed to the increased rate of BA detection from pre to post–NHI years.

In the MHS, where all active duty members and their families have access to medical care, two-thirds of infants with biliary atresia were diagnosed prior to 60 days of life. Despite the economic differences in receiving primary care at a civilian facility versus a military treatment facility, there was no difference in median age at BA diagnosis by access to care models, with timely diagnoses of biliary atresia made at equivalent rates. The median age of diagnosis of BA in the MHS was 40 days of age, ranging from 1 to 189 days, with no significant change in age of diagnosis from 2004 to 2008. Comparing this age of diagnosis to data from the US purchased care system may help uncover any influence healthcare access has on age of diagnosis within this country.

Median age at diagnosis has been recently reported for the 55 patients who underwent a Kasai Procedure for BA at the St Louis Children’s Hospital from 1990–2004 [18]. The overall median age at diagnosis was 60 days, with the breakdown by 5-year time period demonstrating a worsening trend toward increasing age. Median age at diagnosis from 1990 to 1994 was 48.5 days, increasing to 60 days from 1995 to 19999 and 69 days from 2000 to 2004. The difference in median age was significant (p<0.1) comparing those diagnosed from the 1990–1994 period to the more recent 2000–2004 subjects. In 2006, The Biliary Atresia Research Consortium (BARC) studied 104 infants who underwent Kasai Procedure between 1997 and 2000 at 9 clinical centers and demonstrated a mean age at initial evaluation of 53 days, with mean age at surgery of 61 days [19].

Comparing our data to that from within the U.S. purchased care system implies that BA is diagnosed at an earlier age in the MHS. However, there are a number of differences between these studies that make a direct comparison difficult. For example, the St Louis study defines age at diagnosis as the age that subjects underwent Kasai. In our study, age at diagnosis was set as the first date that an inpatient or outpatient encounter was coded as BA, allowing for the possibility of BA diagnosis before Kasai Procedure (if performed). This may lead to a comparative earlier median age in our study when compared to the St Louis study. On the other hand, both the St Louis study and BARC study looked only at that subset of patients who underwent Kasai. Not all patients with BA get this procedure, particularly those who are diagnosed at an advanced age. As our study includes all patients, regardless of Kasai Procedure, the median age could easily be advanced when compared to studies that exclude non-Kasai patients.

There were several limitations to our study. We relied on health claims data and not direct chart review to identify infants with biliary atresia. We minimized ascertainment bias by using a validated algorithm for the identification of infants with congenital conditions in these health claims data. Despite the relative large size of the identified sample compared with other studies of biliary atresia, the sample size was limited to only detect a significant determinant of an early diagnosis of biliary atresia if the effect size was twice or one-half of the comparison group. There is the possibility that primary care model and the other factors considered in our analysis did significantly impact diagnosis at a level less than the minimum detectable difference.

As the importance of early age at Kasai Procedure has been well-established, age at Kasai Procedure was not the focus of this study. However, a limitation of our study is that the M2 database inpatient procedure codes were significantly lacking, making further analysis of our data by presence or absence of Kasai Procedure not possible. There were other limitations secondary to the M2 database data-mining process. Whereas the BARC study found differences in age at Kasai by race and ethnicity, with non-Hispanic whites being more likely to undergo Kasai by 60 days of life [19], the significant absence of race data in our subject population precluded comparison of age at diagnosis by this variable.

Gender, prematurity or presence of additional anomalies did not affect the timing of BA diagnosis in the MHS. In the BARC study, 26 of the total 104 patients (25%) had additional congenital anomalies identified, 11 of which were identified as having Biliary Atresia Splenic Malformation Syndrome (BASM) [19]. Those with BASM were found to undergo evaluation and Kasai at an earlier age, although the great majority of them had a poor outcome. None of our patients had ICD-9 diagnostic codes for spleen anomalies (i.e. polysplenia or other splenic malformations), implying that there were no cases of BASM in our study population; however this could be the result of incomplete coding. We did not examine mortality data and cannot establish whether or not patients with additional anomalies have a worse prognosis than those without.

We were unable to reliably determine the total number of births within the MHS during our study period, making any attempt at annual incidence or overall incidence inaccurate. The Department of Defense Birth and Infant Health Registry [11] published their total five year rate of BA for 2001–2005 as 1.37 per 10,000, with a total of 65 BA patients over this five year period. Unfortunately, there is no distribution by year in this report. Our data is drawn from FY 2004–2008, with a total of 64 BA subjects. Examining the distribution by FY in Table 2, there is a decreasing number of BA diagnoses with subsequent years, yet we cannot determine if this is a change in overall incidence or a reflection of decreased total births during this time period.

Our study design was not able to make causal inferences about the effect of the determinants considered on timely BA diagnosis. Primary care systems in the U.S. are complex systems with national, family, and individual patient level contributors to outcomes. As such, our study was designed to detect associations only to provide insight into possible causal determinants.

The strengths of this study derive from the large study population in an open access health care system that minimizes economic health care disparities. The population also has the option of having their medical care in 2 parallel systems – one provided by civilian providers and one provided by military providers – that allows additional comparisons by type of access model.

### Conclusion

Timely diagnoses of biliary atresia (BA) are made in an open access medical system with a fully insured population. The majority of infants with biliary atresia are diagnosed prior to 60 days of age, when the success of a Kasai procedure is highest. Military and civilian primary care providers make timely BA diagnoses at equivalent rates within the same open-access system. Presence of prematurity, co-existent congenital anomalies, and gender do not influence the timing of BA diagnoses. Fully insured infants in open-access health care systems appear to be diagnosed approximately 2 weeks earlier than in traditional health care systems. Nevertheless, a sizeable portion of our study population (20%) had diagnosis beyond 90 days of age. Efforts must continue to be made to eliminate delayed diagnosis in order to improve success of Kasai and reduce the necessity of liver transplantation. Further studies directly comparing infants in competing health-care delivery systems should be conducted and efforts made to identify factors that may be contributing to delayed diagnosis in subjects with advanced age at BA diagnosis.

## References

[1] Caton AR, Druschel CM, McNutt LA (2004) The epidemiology of extrahepatic biliary atresia in New York State, 1983–98. Paediatr Perinat Epidemiol 18: 97–105. 14996248 10.1111/j.1365-3016.2003.00536.x

[2] Yoon PW, Bresee JS, Olney RS, James LM, Khoury MJ (1997) Epidemiology of biliary atresia: a population-based study. Pediatrics 99: 376–382. 9041292 10.1542/peds.99.3.376

[3] Mowat AP, Davidson LL, Dick MC (1995) Earlier identification of biliary atresia and hepatobiliary disease: selective screening in the third week of life. Arch Dis Child 72: 90–92. 7717750 10.1136/adc.72.1.90 PMC1510987

[4] Hadzic N, Davenport M, Tizzard S, Singer J, Howard ER, et al (2003) Long-term survival following Kasai portoenterostomy: is chronic liver disease inevitable? J Pediatr Gastroenterol Nutr 37: 430–433. 14508212 10.1097/00005176-200310000-00006

[5] Chardot C, Carton M, Spire-Bendelac N, Le Pommelet C, Golmard J, et al (2001) Is the Kasai operation still indicated in children older than 3 months diagnosed with biliary atresia? J Pediatr 138: 224–228. 11174620 10.1067/mpd.2001.111276

[6] Mieli-Vergani G, Howard ER, Portman B, Mowat AP (1989) Late referral for biliary atresia–missed opportunities for effective surgery. Lancet 1: 421–423. 2563796 10.1016/s0140-6736(89)90012-3

[7] Moyer V, Freese DK, Whitington PF, Olson AD, Brewer F, et al (2004) Guideline for the evaluation of cholestatic jaundice in infants: recommendations of the North American Society for Pediatric Gastroenterology, Hepatology and Nutrition. J Pediatr Gastroenterol Nutr 39: 115–128. 15269615 10.1097/00005176-200408000-00001

[8] Bibb SC (2001) The relationship between access and stage at diagnosis of breast cancer in African American and Caucasian women. Oncol Nurs Forum 28: 711–719. 11383185

[9] Farley JH, Hines JF, Taylor RR, Carlson JW, Parker MF, et al (2001) Equal care ensures equal survival for African-American women with cervical carcinoma. Cancer 91: 869–873. 11241257

[10] Thomas AG, Brodine SK, Shaffer R, Shafer MA, Boyer CB, et al (2001) Chlamydial infection and unplanned pregnancy in women with ready access to health care. Obstet Gynecol 98: 1117–1123. 11755563

[11] Population-based Birth Defects Surveillance data from selected states, 2001–2005. Birth Defects Res A Clin Mol Teratol 82: 831–961. 10.1002/bdra.20549 19072842

[12] Chardot C, Serinet MO (2006) Prognosis of biliary atresia: what can be further improved? J Pediatr 148: 432–435. 16647399 10.1016/j.jpeds.2006.01.049

[13] Sokol RJ, Shepherd RW, Superina R, Bezerra JA, Robuck P, et al (2007) Screening and outcomes in biliary atresia: summary of a National Institutes of Health workshop. Hepatology 46: 566–581. 17661405 10.1002/hep.21790 PMC3888317

[14] Hsiao CH, Chang MH, Chen HL, Lee HC, Wu TC, et al (2007) Universal screening for biliary atresia using an infant stool color card in Taiwan. Hepatology 47: 1233–1240. 10.1002/hep.22182 18306391

[15] Matsui A, Ishikawa T (1994) Identification of infants with biliary atresia in Japan. Lancet 343: 925. 10.1016/s0140-6736(94)90052-3 7908393

[16] Cheng TM (2003) Taiwan’s new national health insurance program: genesis and experience so far. Health Aff (Millwood) 22: 61–76. 12757273 10.1377/hlthaff.22.3.61

[17] Chen CS, Liu TC (2005) The Taiwan National Health Insurance program and full infant immunization coverage. Am J Public Health 95: 305–311. 15671469 10.2105/AJPH.2002.012567 PMC1449171

[18] Wadhwani SI, Turmelle YP, Nagy R, Lowell J, Dillon P, et al (2008) Prolonged neonatal jaundice and the diagnosis of biliary atresia: a single-center analysis of trends in age at diagnosis and outcomes. Pediatrics 121: e1438–1440. 18443020 10.1542/peds.2007-2709

[19] Shneider BL, Brown MB, Haber B, Whitington PF, Schwarz K, et al (2006) A multicenter study of the outcome of biliary atresia in the United States, 1997 to 2000. J Pediatr 148: 467–474. 16647406 10.1016/j.jpeds.2005.12.054

